# Impact of Green Extraction Methods for Algae and Aquatic Plants on Amino Acid Composition and Taste Detection Using Electronic Tongue Analysis

**DOI:** 10.3390/foods15020305

**Published:** 2026-01-14

**Authors:** Lyket Chuon, Witoon Prinyawiwatkul, Amporn Sae-Eaw, Peerapong Wongthahan

**Affiliations:** 1 Department of Food Technology, Faculty of Technology, Khon Kaen University, Khon Kaen 40002, Thailand; lyket.c@kkumail.com (L.C.); sampor@kku.ac.th (A.S.-E.); 2 School of Nutrition and Food Sciences, Agricultural Center, Louisiana State University, Baton Rouge, LA 70803, USA; wprinya@lsu.edu

**Keywords:** maceration extraction, ultrasound-assisted extraction, enzyme-assisted extraction, umami, seaweed, freshwater algae, e-tongue

## Abstract

The growing demand for sustainable protein sources has increased interest in algae and aquatic plants as alternatives to animal-derived proteins. These resources are rich in protein, amino acids, and umami compounds but require suitable extraction methods to maximize yield and quality. This study compared three green extraction techniques—maceration (MAE, 80 °C, 2 h), ultrasound-assisted extraction (UAE, 750 W, 20 kHz, 50% amplitude, 35 °C, pH 12, 1 h), and enzyme-assisted extraction (EAE, 5% β-glucanase/flavourzyme, 55 °C, pH 6.5, 1 h)—on five raw materials: wakame (commercial seaweed), hair seaweed, sea lettuce, water silk algae, and *Wolffia*. The result revealed that both raw materials and extraction methods significantly (*p* < 0.05) affected protein yield, amino acid, physicochemical properties, and taste detection with e-tongue. *Wolffia* extracted by MAE yielded the highest protein overall, followed by UAE and EAE methods, when compared with commercial seaweed. The relationship between amino acid profiles and taste detection was investigated by principal component analysis (PCA) and hierarchical cluster analysis (HCA); the samples with higher glutamic and aspartic acids were linked with umami taste, while histidine contributed to bitter taste. Overall, the findings highlighted that extraction efficiency was influenced more by the extraction method–material compatibility than the raw material alone.

## 1. Introduction

According to Food and Agriculture Organization (FAO), the global population is predicted to reach 9.7 billion by 2050, requiring a nearly 60% increase in food production to meet the growing demand [1]. Therefore, many studies are focused on finding healthier food sources to address these issues. The livestock sector contributes to 30% of biodiversity loss and 14.5% of global GHS emissions [2]. Moreover, one billion people worldwide lack access to affordable and wholesome food, and excessive meat consumption as a protein source is seen as a major issue for climate change and sustainable production practices. Thus, sustainable production methods are necessary to reduce the carbon footprint [3,4].

Recently, there has been drastic interest in plant-based proteins as potential substitutes for animal proteins. These proteins can be produced more efficiently and with a lower environmental impact than animal proteins, offer a promising solution to future food security challenges and lack of protein intake, while also providing several potential health benefits [5]. Alongside this trend, there has also been increased interest in umami [6,7,8,9,10,11], because of its unique nutritional and health benefits [12]. Umami has been recognized as one of the five fundamental tastes that contributes to a savory and rich flavor to a variety of dishes. Its characteristic taste is primarily attributed to compounds such as monosodium glutamate (MSG), free amino acids (FAAs) like aspartic acid (Asp) and glutamic acid (Glu), and 5′-mononucleotides such as 5′-guanosine monophosphate (GMP), 5′-inosine monophosphate (IMP), and 5′-xanthosine monophosphate (XMP) [13].

Algae should be recognized and valued more widely as promising alternative protein sources that can drive meaningful changes in sustainable food systems. They are abundant, protein-rich organisms that are found in both freshwater (called freshwater algae) and marine habitats (called seaweed) [14]. The crude protein content in freshwater macroalgae ranged from 16 to 44% of dry weight [15], while seaweeds contain about 5 to 47% of dry weight [16], making them promising sources of protein. Notably, the protein content (10–47%) in green and red seaweeds is generally higher compared to that in brown seaweeds (5–25% dry weight) [17]. For example, the green algae like water silk algae (*Spirogyra* spp.) can reach 22.77% [18], while wakame (*Undaria pinnatifida*) typically contains around 18.0% [19]. Similarly, species of the genus *Wolffia*, including *Wolffia arrhiza*, have been reported to contain approximately 20–30% crude protein on a dry weight basis, depending on species and cultivation conditions, highlighting their potential as protein-rich freshwater biomass for food applications [20]. In particular, the *Wolffia arrhiza* is traditionally consumed as a culinary ingredient in the northeast and north of Thailand [21].

In addition to total protein, several studies have highlighted that amino acid composition of algae such as sea lettuce (*Ulva lactuca*), water silk algae (*Spirogyra neglecta*), and other edible seaweeds are rich in amino acids, particularly aspartic and glutamic acids, which are the key contributors to umami taste [22,23]. Algae are also rich in minerals, vitamins, dietary fiber, fatty acids, polysaccharides, and bioactive molecules with a wide range of therapeutic potential that could further enhance human well-being and promote a balanced diet when regularly consumed. Furthermore, algae have unique technological qualities that enable their incorporation into dairy, fish, meat, mashed potatoes, fish cakes, and pasta products, among others, preserving or enhancing their sensory, nutritional, and health-promoting advantages [6,24,25].

There are several methods to extract protein and derivatives, such as amino acid or umami compounds, from algae. Therefore, it is essential to identify and use the method that is novel and effective for recovering bioactive compounds found in algae. Maceration extraction (MAE) can offer the benefits of simple use, inexpensiveness, and environmental friendliness [26]. Ultrasound-assisted extraction (UAE) is considered one of the most important technologies for achieving the goals of sustainable extraction. It is a non-thermal method that generates significant shear forces in the extracting medium [27]. UAE uses the high-frequency sound waves, typically between 20 kHz and 100 MHz, to break down plant materials. These waves create tiny bubbles that rapidly collapse, generating shockwaves that disrupt cell walls and help release bioactive compounds. This technique is widely appreciated for its ability to extract bioactive ingredients efficiently while being easy to operate, cost-effective, and speeding up the extraction process [28]. Enzyme-assisted extraction (EAE) is a process where intracellular molecules are released into a solvent when enzymes break down the plant cell wall. The efficiency of the extraction largely depends on the type of hydrolytic enzymes used. Moreover, the optimal enzyme activity is strongly influenced by factors such as pH, temperature, and reaction time [29]. These green extraction methods do not only improve protein recovery but also support global sustainability, especially SDG 2 (Zero Hunger), SDG 12 (Responsible Consumption and Production), and SDG 13 (Climate Action) [30].

Therefore, this study was aimed at investigating the physicochemical properties and the taste profiles of the protein extracted from algae and plant using green extraction methods, namely MAE, UAE, and EAE. Based on this, the following hypotheses were proposed:

**H1:**  *The raw materials do not affect the proximate composition.*

**H2:**  *The green extraction methods do not affect the physicochemical properties of the protein extracts.*

**H3:**  *The green extraction methods do not affect the taste profile of the protein extracts as determined by the e-tongue.*

**Figure 1:**
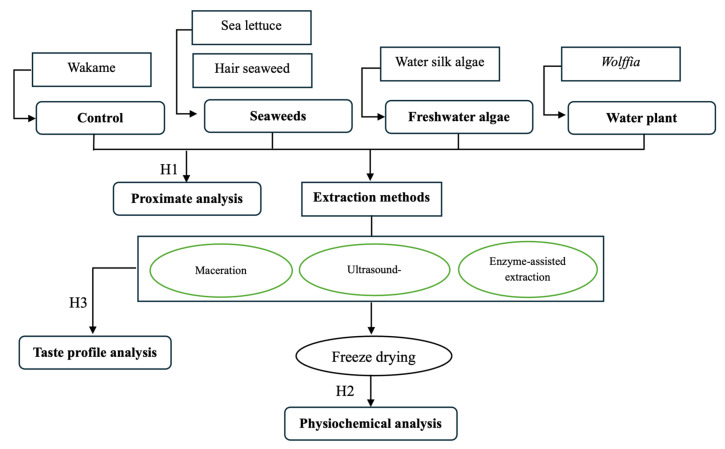
The outline of the research and the proposed hypotheses.

Figure 1 presents a schematic overview of the experimental design and the relationships among raw materials, extraction methods, and analytical approaches used in this study. The selected aquatic materials were grouped into commercial control, seaweeds, freshwater algae, and water plant.

## 2. Materials and Methods

### 2.1. Raw Material Preparation

Five dried raw materials were used in this experiment (Table 1): wakame (*Undaria pinnatifida* (Harvey) Suringar, 1873) was selected as a control sample (K J C Interfood Company Limited, Bangkok, Thailand); hair seaweed (*Gracilaria fisheri* (B.M.Xia and I.A.Abbott) I.A.Abbott and J.N.Norris 1991, Yo Island, Song Khla province, Thailand) and sea lettuce (*Ulva lactuca*, L., Species Plantarum 2: 1163 (1753), Leam Sing district, Chanthaburi province, Thailand) represented the seaweed category; water silk algae (*Spirogyra neglecta* (Hassall) Kützing 1849, Fresh water processing community enterprise, Nan province, Thailand) represented a freshwater algae; and *Wolffia* (*Wolffia arrhiza* L. Horkel ex Wimm. 1857, Vittoon Farm, Pitsanulok province, Thailand) represented a water plant. All samples were collected from January to July 2024. The obtained dried materials were initially ground using a high-speed grinder (WJX 1500A, Shanghai Yuanlu Wugong Co., Ltd., Shanghai, China) followed by further grinding with Ultra Centrifugal Mill (Retsch ZM200, Retsch GmbH, Retsch-Allee 1-5 42781 Haan, Germany). The samples were then sieved through a 500 μm mesh (sieve No. 35) to produce a fine uniform powder. The powdered samples were stored in vacuum-sealed bags and kept refrigerated for subsequent analysis. The samples used in this study were produced in compliance with Thai FDA standards, which regulate heavy metal content, physiochemical properties, and microbiological quality. All measure parameters were within the acceptable limits specified by food safety regulations.

### 2.2. Chemicals and Commercial Enzymes

All chemicals used for analysis were analytical grade except for HCl acid and NaOH, which were food grade and used for the extraction process. The commercial enzymes used in the experiment included β-glucanase, produced by fermentation of *Aspergillus niger* with a minimum activity of 5000 U/g, and flavourzyme, derived from *Aspergillus oryzae* with a minimum activity of 500 LAPU/g. Both commercial enzymes were purchased from Chanchao Longevity Co., Ltd., Bangkok, Thailand.

### 2.3. Proximate Analysis of Raw Materials

The proximate composition including moisture, ash, protein, crude fiber, and crude fat was determined in triplicate using the standard AOAC methods 925.10, 942.03, 950.52, 978.10, and 963.39, respectively [31]. Carbohydrate content was calculated by the difference.

### 2.4. Protein Powder Extraction

The extraction methods were applied under their respective optimized conditions, following literature and preliminary optimization, rather than under identical extraction parameters for direct comparison. The ratio of dried raw materials to distilled water was 1:20 (*w*/*v*) for all extractions.

- Maceration extraction (MAE) was carried out with modifications based on the method described by Ummat et al. [11]. Initially, distilled water was preheated to 80 °C and then mixed with the dried raw material powder. The extraction was performed in a water bath (WNB22, Memmert, Schwabach, Germany) maintained at 80 °C and stirred at 200 rpm for 2 h. The extract was centrifuged at 9190× *g* (Hitachi Koki/model CR22N/himac Rotor R15A, Tokyo, Japan) for 20 min at 4 °C. The pH of each extraction solution was slightly acidic to close to neutral, ranging from 5.86 to 6.94.
- Ultrasound-assisted extraction (UAE) was performed using an ultrasonic processor (model VCX 750, Sonic & Materials, Inc., Newtown, CT, USA) equipped with a 25 mm diameter probe. This method was modified from Purdi et al. [32]. The sonicator was operated at 750 W power and a frequency of 20 kHz. To maintain the extraction temperature below 35 °C, an ice bath was employed during sonication. The process was operated by setting the ultrasonic amplitude to 50%, setting the time at 1 h and a duty cycle of 60% (pulsing 3 s on, 2 s off). The solution’s pH was adjusted to 12 using 2 M NaOH. The ultrasonic probe was vertically positioned at the center of the vessel, with the tip immersed approximately 2 cm above the bottom, ensuring efficient cavitation. Following sonication, the mixture was centrifuged at 9190× *g* for 20 min at room temperature. The pH of the supernatant was then adjusted to the isoelectric point (pI) of the individual sample as presented in Table 2 using 2 M HCl, facilitating protein precipitation. The solution was left in a cooling bath at 10 °C for 30 min, followed by another centrifugation step (25 min, 11,600× *g*, room temperature). Finally, the precipitate was collected and neutralized to pH 7.0 by dissolving the pellet with the distilled water until the final volume of 500 g, corresponding to 50 g of raw material.
- Enzyme-assisted extraction (EAE) was conducted following a procedure adapted from Poojary et al. [10]. The pH of the solvent was carefully maintained at 6.5 by adjusting with 1 M of HCl or NaOH as needed during the extracting process. A mixture of 1:1 (*w*/*w*) enzymes of β-glucanase and flavourzyme was added to facilitate extraction. Extraction was performed at 55 °C in a water bath with an enzyme concentration set at 5% (*w*/*w*), based on the conditions reported by Rodrigues et al. [33]. The process continued for 1 h. To inactivate the enzyme activity, the mixture was heated at 90–100 °C for 15 min. Afterwards, the solution was cooled in a cooling bath before centrifugation at 9190× *g* for 20 min at room temperature to separate the solid residue from the extract. The pH of the solution obtained from each raw material was 6.5 after centrifugation.

The resulting supernatant from each material and extraction method was collected and freeze-dried (Christ, Model Beta 1-8 LSCplus, Osterode am Harz, Germany). The freeze-dried samples were ground by using a high-speed grinder (WJX 1500A, Shanghai Yuanlu Wugong Co., Ltd., Shanghai, China) to produce the fine powder. The powder was packed into the aluminum vacuum seal and stored in the refrigerator for further analysis.

**Table 2:** The isoelectric point of different raw materials.

Protein Sources	Isoelectric Point	References
Wakame	2	[34]
Hair seaweed	4.3	[35]
Sea lettuce	2	[36]
Water silk	3	Our preliminary study
*Wolffia*	3.5	[37]

### 2.5. Physicochemical Analysis of Protein Extract Powders

Moisture and water activity content were conducted following the AOAC (2000) procedures [31]. Salt content was measured using the Mohr’s titration method adapted from Susi and Al Hakim. [38]. Protein content was determined using the Lowry’s method [39]. Color measurement was performed by colorimetry (HunterLab, MiniScan EZ System, Reston, VA, USA) based on the CIELAB color (L*, a*, and b*), according to Chen et al. [40]. The white index was calculated by following [41]:

(1) $$\mathrm{WI}\;=\;100\;-\;\sqrt{{(100\;-{\;L}^{*})}^{2}{+\;(a}^{*}{)}^{2\;}{+\;(b}^{*}{)}^{2}}$$

The amino acid analysis was conducted by the Northern Science Park, Khon Kaen University, Khon Kaen, Thailand, according to the method from ISO 13903 [42]. All samples were hydrolyzed in DigiTubes using HCl 6 mol/L at 110 °C for 22 h. In total, 16 amino acids were measured, except Met and Cys as both were partially destroyed during the acid hydrolysis step. The content of amino acids was determined using an ion-exchange chromatography (in a 150 mm × 4.6 mm Cation Separation Column, K06/Na) with a post-column derivatization and spectrophotometric detection of ninhydrin reaction products, and with the use of an automatic amino acid analyzer (SCION Artimis 6000 Amino Acid Analyzer, Goes, The Netherland), according to the manufacturer’s standard procedure. The flow rate of the eluent was set to 0.45 mL/min, with an injection volume of 20 μL. The reactor was operated at 130 °C with a flow rate of 0.25 mL/min, and detection was performed using a UV/Vis detector at wavelengths of 470 nm and 570 nm.

### 2.6. E-Tongue Taste Profile Analysis

Taste profiles were performed in triplicate using an e-tongue system (TS-5000Z, Intelligent Sensor Technology Inc., Atsugi City, Japan), comprising multichannel lipid/polymer membrane sensors, a data acquisition unit, an auto sampler, reference electrodes, with sensor output data exported for chemometric analysis using XLSTAT 2025.1.3 (1431) to enable combined analysis with amino acid composition. Each sample was measured in four steps; the first measurement was discarded as a stabilization step, and the remaining steps were averaged and used for data analysis. An Ag/AgCl reference electrode was used to record potentiometric responses, calculated as the voltage difference between each sensor and the reference electrode once equilibrium in standard solutions had been reached. The protein extract solutions of each extraction method were used to analyze for their taste profiles. Prior to measurement, pH and conductivity were checked. The system assessed 6 sensors (AAE, CT0, CA0, UM2, C00, and AE1), which can detect tastes such as saltiness, sourness, umami, bitterness, astringency, umami aftertaste, bitter aftertaste, and astringent aftertaste. Sweetness was not measured, as the TS-5000Z cannot detect sweetness, including that from amino acids in the protein extracts. The e-tongue analytical procedure is described in detail in the Supplementary Materials.

### 2.7. Statistical Analysis

The entire experiment was performed in triplicate. Proximate composition data were reported as mean ± standard deviations and analyzed using IBM^®^ SPSS Statistics (Version 29.0.2.0 (20)). Differences among raw materials and extraction methods in protein content and physicochemical properties of protein powder extracts (moisture, water activity, color, and salt content) were determined using the analysis of variance (ANOVA). Duncan’s multiple range test was performed as a post hoc test when data showed a significant difference at *p* < 0.05. Principle component analysis (PCA) and hierarchical cluster analysis (HCA) were performed on the taste profiles and amino acids of each protein sources from different extraction methods using XLSTAT 2025.1.3 (1431).

## 3. Results

### 3.1. Proximal Composition of Protein Sources

The proximate analysis of five protein sources was summarized in Table 3, which shows the significant differences (*p* < 0.05) among the protein sources. The protein content of wakame was the lowest among the samples (15.20%), which was similar to the value of 16.8% previously reported by Taboada et al. [38]. In contrast, sea lettuce showed the highest protein content (25.70%), aligned with that (24.17%) reported by Peter et al. [43]. Among the samples, sea lettuce had the highest protein content, followed by hair seaweed, water silk algae, and *Wolffia*, while wakame showed the lowest protein content and crude fiber content. Wakame and water silk had a much higher ash content (30.12–31.29%) compared to the rest (7.17–14.95%) (Table 3). For wakame, comparable ash values have been reported in the literature (28.3%) [44]. In contrast, the ash content of water silk algae (*Spirogyra neglecta*) reported by Yongkhamcha and Buddhakala [45] was lower (10.73%), suggesting that ash content may vary substantially depending on growth environment, water mineral composition, and processing conditions.

### 3.2. Physicochemical Analysis of Protein Extracts from Different Extraction Methods

#### 3.2.1. Moisture Content and Water Activity

The moisture content and water activity (a_w_) were significantly different (*p* < 0.05) among the samples, which ranged from 0.64 ± 0.10% to 8.82 ± 0.03% and 0.10 ± 0.01 to 0.38 ± 0.01, respectively (Table 4). Both moisture content and water activity (a_w_) parameters are crucial for ensuring the stability of the product during storage, especially before further utilization in required applications. All extract samples (Table 4) had both moisture content and water activity below the recommended values of 10% and 0.60–0.65 [46]. The products with higher moisture and water activity (a_w_) tend to deteriorate more quickly, as these parameters significantly affect microbial growth, lipid oxidation, and both enzymatic and non-enzymatic reactions in the food systems [47,48].

#### 3.2.2. Color

**Figure 2:**
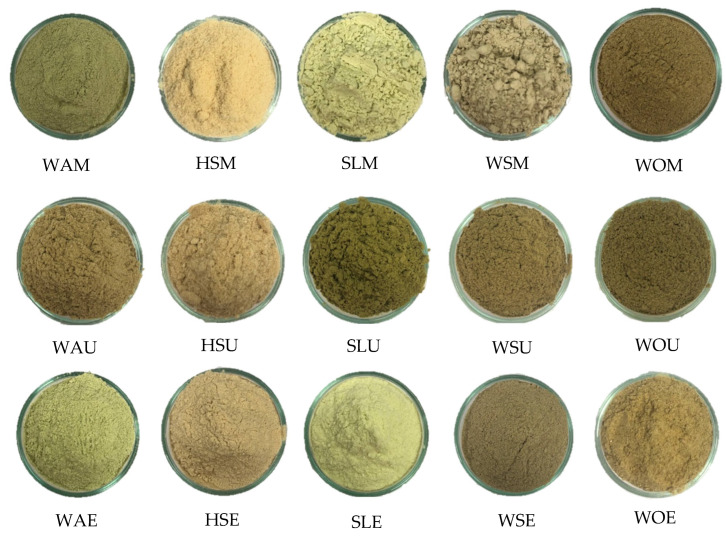
The protein extract powders from five raw materials and three extraction methods. See Table 1 for sample abbreviations and their descriptions.

Among the quality attributes, color is one of the most important attributes consumers readily notice, as it affects the appearance of food [49]. Color was measured using the CIELAB color system. In this system, L* indicates lightness, ranging from black (L* = 0) to white (L* = 100). The a* value represents the red–green axis, where positive values indicate redness and negative values indicate greenness. The b* value represents the yellow–blue axis, with positive values indicating yellowness and negative values indicating blueness. Under the MAE and EAE methods, sea lettuce had the highest L*, while both wakame and sea lettuce showed higher negative a* values. The white index of the dried protein extracts was measured [WI = 100 indicates “very close to pure white”]. The WI among samples were significantly different across the three extraction methods (*p* < 0.05). The values ranged from 48.60 ± 0.24 to 67.29 ± 0.42 for MAE, and 53.61± 0.11 to 67.35 ± 0.08 for EAE, which were brighter than UAE with WI ranging from 39.36 ± 0.42 to 56.69 ± 0.56 (Table 4). The color of the five samples across three extraction methods can be visualized in Figure 2. Under the MAE and EAE methods, the sea lettuce (SLM and SLE) exhibited lighter and more vivid green color compared to others (Figure 2 and Table 4). Based on the study of Soimaloon et al. [50], color can be affected by many factors, such as inherent natural pigment, pH, temperature, and time during the extraction process. Similarly, other studies have reported that pigment stability in algae and plant extracts is highly influenced by processing conditions, particularly pH and heat [51,52].

#### 3.2.3. Salt and Protein Contents

The salt content of the protein extracts with the different extraction methods was measured to assess its impact on the quality and practical use of the extracts. The highest level of salt content was observed in wakame across all three extraction methods, MAE, UAE, and EAE, at 21.56 ± 2.09%, 8.80 ± 0.24%, and 24.18 ± 0.46%, respectively (Table 5). This could be due to the naturally high salt content in wakame which may also help regulate the pH during the extraction process. In the case of hair seaweed, a notably higher salt content was observed after UAE compared to MAE and EAE. This occurred because during the pH adjustment to the pI, the protein pellet was not able to be isolated as anticipated. Consequently, the pH was directly adjusted to neutral, which resulted in a high salt content in the final sample. According to Torres et al. [53], the genus of *Gracilaria* including hair seaweed contains a high level of agar, which may pose a significant barrier to protein precipitation, as the gel-forming polysaccharides in *Gracilaria* can interfere with the isolation of the protein pellet during extraction.

The protein content in different samples was analyzed using Lowry’s method, as shown in Table 5. Among all the tested raw materials, *Wolffia* consistently exhibited the markedly highest protein yield, particularly under MAE, reaching 100.79 ± 5.28 mg/g. Other samples contained less protein: water silk algae (24.25 ± 2.19 mg/g), hair seaweed (8.86 ± 0.20 mg/g), wakame (5.90 ± 0.32 mg/g), and sea lettuce (2.30 ± 0.25 mg/g). Interestingly, *Wolffia* also maintained relatively high yields under UAE (51.95 ± 2.66 mg/g) and EAE (51.88 ± 0.64 mg/g), although both were significantly (*p* < 0.05) lower than MAE. The exceptionally high protein yield from *Wolffia* under MAE may be attributed to its thin and non-rigid cell walls and naturally soluble proteins, which allow efficient extraction without the need for disruptive treatments [54]. More intensive techniques, such as UAE or EAE, may disrupt additional cellular components or dilute protein fractions, thereby reducing recovery [55].

UAE also produced notably high protein levels in *Wolffia* (51.95 ± 2.66 mg/g), the highest among all UAE-tested samples (Table 5), even though the protein content from *Wolffia*’s raw protein content was not the highest (Table 3). Moreover, UAE proved particularly effective for sea lettuce, yielding 7.32 ± 0.56 mg/g, which was significantly higher than MAE (2.30 ± 0.25 mg/g) and EAE (5.95 ± 0.45 mg/g). The improvement from UAE can be explained by the cavitation effect of ultrasound, which generates shear forces and micro-jets that disrupt robust cell walls, enhancing protein release [56] from species with rigid cell structures [57]. For hair seaweed, UAE and MAE were not significantly different (*p* > 0.05) in the protein content but outperformed EAE. This suggests that the barrier to protein release may not be limited to cell wall toughness but also the presence of different types of thick polysaccharides (e.g., alginate, carrageenan, ulvans) that trap proteins within the cell matrix [58,59,60].

EAE showed variable effects among the tested algae. In hair seaweed, the protein yield obtained by EAE (1.55 ± 0.20 mg/g) was significantly lower than that achieved through MAE (8.86 ± 0.20 mg/g) and UAE (8.98 ± 0.43 mg/g) (Table 5). This suggests that enzymatic treatment alone was not sufficient to disrupt its rigid structure. Hair seaweed (*Gracilaria* spp.) contains a high proportion of sulfated galactans such as agar and carrageenan, which form strong gel networks and increase cell wall rigidity [61]. These polysaccharides act as physical barriers, limiting enzyme accessibility and protein release. For wakame, its yield was 4.33 ± 0.19 mg/g, which was lower than MAE (5.90 ± 0.32 mg/g). De Souza Celente et al. [62] did not recommend enzymatic hydrolysis for brown seaweeds, as their cell wall complexity, which is rich in cellulose, glucan, alginic acid, sulfated xyloglucan, sulfated xylofucoglucuronan, alginate, fucoidan, laminarin, and mannitol, makes them less susceptible to enzymatic degradation. In contrast, the study of Naseri et al. [63] evaluated different commercial enzymes for protein extraction from *Palmaria palmata*, and reported that proteases such as Alcalase and Flavourzyme achieved extraction yields above 90% under optimized conditions, while cellulase and other carbohydrases facilitated additional release of proteins and amino acids, acting specifically based on the composition of the seaweed cell wall. Malvis Romero et al. [64] highlighted that enzyme selection was guided by the structural components of *Ulva fenestrata*, such as the presence of ulvan and cellulose, to enhance extraction efficiency and obtain targeted bioactive compounds. Overall, the success of EAE is highly dependent on seaweed type and strongly influenced by enzyme type and its specificity toward the structural components of the seaweed cell wall.

Overall, the findings suggest that MAE is a more efficient method for extracting proteins, especially from the aquatic plant (*Wolffia*) and freshwater algae (water silk). Therefore, selecting the appropriate extraction method and/or enzyme based on the specific material composition is crucial.

#### 3.2.4. Amino Acid Content

The amino acid analysis is presented in Table 6. All samples contained Asp (aspartic acid) and Glu (glutamic acid), which are key contributors to umami flavor; however, their concentrations varied depending on the raw material and the extraction method employed. Notably, *Wolffia* samples obtained via maceration exhibited the highest levels of Asp (20.26 × 10^−2^ g/100 g) and Glu (21.70 × 10^−2^ g/100 g), much greater than other samples. Furthermore, samples extracted using ultrasound-assisted extraction (UAE) yielded even higher concentrations of these amino acids in *Wolffia*, with Asp and Glu contents reaching 26.21 × 10^−2^ g/100 g and 31.20 × 10^−2^ g/100 g, respectively. These results suggest that UAE enhances amino acid release, potentially due to cavitation-induced disruption of cell walls, which facilitates improved solvent diffusion and extraction efficiency [27]. For enzyme-assisted extraction, *Wolffia* maintained elevated levels of Asp (19.82 × 10^−2^ g/100 g) and Glu (20.64 × 10^−2^ g/100 g), confirming its stability across methods. Water silk also provided moderate concentrations but remained lower than *Wolffia* under all conditions tested. Overall, the commercial wakame had the lowest Asp and Glu compared to the two freshwater materials, *Wolffia* and water silk (Table 6).

#### 3.2.5. Taste Profiles by Electronic Tongue

The electronic tongue results showed clear differences among the algae extracts as shown in Table 7. The strong umami intensity was found in water silk and *Wolffia* across the extraction methods (WOM, WSM, WOU, WSU, WOE, and WSE) while SLU had the lowest intensity. The umami aftertaste was also higher in WOM and WOE, indicating a longer-lasting umami sensation. For bitterness, the highest values appeared in WSU and HSU, which may be due to co-extracted bitter peptides or phenolic compounds [65]. Meanwhile, WOE and WOM showed very low bitterness. A strong salty taste was also detected in WSM and WSE, reflecting the natural mineral content of algae. In sum, the e-tongue confirmed that algae extracts have complex taste profiles dominated by umami, salty, and bitter notes. Optimizing extraction conditions could maximize desirable umami intensity and persistence while minimizing off flavors (such as bitter taste), with sensory validation and chemical profiling recommended to identify the responsible compounds.

### 3.3. Relationship Among Amino Acids and Taste Profiles Using Principal Component Analysis (PCA)

**Figure 3:**
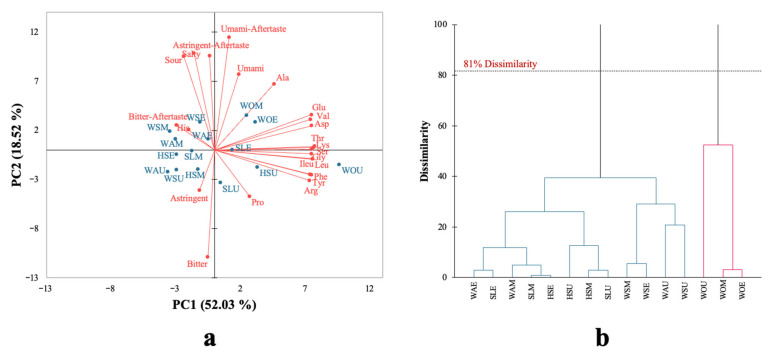
Principal component analysis (PCA) (**a**) and Hierarchical cluster analysis (HCA) (**b**) of protein extracts from 5 raw materials across three extraction methods based on their amino acid composition and taste profiles from e-tongue.

The relationships among raw materials from three extraction methods (maceration extraction, ultrasound-assisted extraction, and enzyme-assisted extraction) (see Table 1) with amino acids and taste profiles obtained from e-tongue analysis are presented as PCA biplots in Figure 3a. The two main components together explained 70.55% of the total variability with PC1 accounting for 52.03% and PC2 for 18.52%. For PC1, results showed a correlation among the taste of umami, umami aftertaste, and amino acids (including Glu, Asp, Ala, Val, Thr, Lys, Ser, Gly, Ileu, and Lue) were strongly associated with six samples (WOM, WOE, WOU, SLE, HSU, and SLU). On the other hand, the tastes of salty, sour, astringent, astringent aftertaste, bitter, bitter aftertaste, and the amino acid (His) were associated with nine samples (WSE, WSM, WAE, WAM, WAU, SLM, HSE, HSM, and WSU), as indicated by the factor loadings for these taste attributes of −0.207, −0.302, −0.147, −0.374, and −0.108, respectively. Moreover, hierarchical cluster analysis (HCA) was performed to classify the group of samples according to their characteristics; at around 81% of dissimilarity the protein powder extracts were classified into two groups (Figure 3b). This separation suggests that *Wolffia* extracts contained higher levels of umami-related amino acids, especially Glu and Asp, which are widely recognized as the main contributors to umami taste. This trend may be related to the highest protein content obtained by Lowry method, as *Wolffia* consistently gave the highest protein yield among all raw materials and extraction methods (Table 5). Similar results were reported in other studies, with seaweed samples based on their amino acid composition (Glu and Asp) as the main drivers of umami flavor [7]. Although *Wolffia* is not a seaweed but an aquatic plant [66], the presence of umami-related amino acids in its protein extracts can explain the observed umami response. While umami taste is commonly associated with seaweed, it is not unexpected in *Wolffia* extracts when sufficient levels of glutamic and aspartic acids are present. Interestingly, the sample clustered on the left side of PC1 was more linked to bitter and astringent characteristics, which is consistent with findings reported in mushroom products, where elevated amino acid (His) was correlated with bitterness [67]. In addition to amino acid levels, the relative proportions of umami- and bitter-related amino acids can also influence taste perception. Metrics such as the umami-to-bitter amino acid ratio may therefore provide additional insight into how different extraction methods affect the taste characteristics of the extracts. These results also indicate that the extraction methods influenced the taste attributes, particularly WSE, WAE, and HSE from enzyme-assisted extraction showing the strong correlation with bitter taste and bitter aftertaste. This agrees with the previous studies reporting that enzymatic hydrolysis can generate bitter peptides or free amino acids [68,69]. Overall, the total amino acid content from green extraction techniques, such UAE and EAE, was higher than that from maceration extraction, demonstrating how well green extraction can work to extract protein-bound amino acids from raw materials (Table 6). These findings are consistent with previous research reporting that the yield of amino acid extracts depends on the extraction methods, extraction conditions, and different materials [7,11,70].

## 4. Conclusions

This study evaluated the influence of different extraction methods [maceration extraction (MAE), ultrasound-assisted extraction (UAE), and enzyme-assisted extraction (EAE)] on extraction yield and physicochemical properties of protein extract powders prepared from five different materials [seaweeds, freshwater algae, and aquatic plant] in Thailand. Results revealed that the type of raw materials and extraction methods employed significantly impacted the protein yield. Notably, *Wolffia*, with its soft cell walls, provided the highest protein yield, while seaweeds showed lower protein due to their complex polysaccharide-rich cell wall structures. Importantly, the extractable protein yield was not always compatible with the protein content in the raw material, highlighting the importance of raw material composition, extraction methods and conditions. In this study, the UAE offered a higher yield of umami-enhancing glutamic and aspartic acids in some samples. A strong correlation between amino acid composition and e-tongue analysis demonstrated that glutamic and aspartic acids were key contributors to umami taste, confirming a clear link between amino composition and taste detection. The findings highlighted the critical role of extraction methods for the nutritional value and sensory detection of the extracts, as they had significant effects on protein yields, amino acid composition, and associated taste attributes. It should be noted that the extraction methods were evaluated under their respective optimized conditions and were not intended for direct one to one comparison under identical parameters. Future research should explore optimized extraction methods and conditions for each raw material, considering their unique cell wall composition to maximize protein yield, improve protein functionality, protein structure (SDS-PAGE profiling) and enhance taste-active compounds. In addition, future studies should incorporate the simultaneous quantification of 5′-mononucleotides (e.g., 5′-GMP, 5′-IMP, 5′-XMP) alongside free amino acids to provide a more comprehensive characterization of umami-related components in extracts derived from algae and aquatic plants.

## Figures and Tables

**Table 1:** Five types of protein sources used in this study.

Raw Materials	Extraction Methods		
	Maceration Extraction (M)	Ultrasound-Assisted Extraction (U)	Enzyme-Assisted Extraction (E)
Wakame (WA)	WAM	WAU	WAE
Hair seaweed (HS)	HSM	HSU	HSE
Sea lettuce (SL)	SLM	SLU	SLE
Water silk (WS)	WSM	WSU	WSE
*Wolffia* (WO)	WOM	WOU	WOE

**Table 3:** Proximate composition * of the protein sources (% dry weight).

Sample	Moisture	Ash	Fat	Protein	Crude Fiber	Carbohydrate
Wakame	9.69 ± 0.03 ^b^	30.12 ± 0.01 ^b^	1.29 ± 0.10 ^c^	15.20 ± 0.11 ^e^	3.21 ± 0.08 ^e^	40.49 ± 0.25 ^d^
Hair seaweed	8.29 ± 0.09 ^c^	7.17 ± 0.01 ^e^	0.05 ± 0.07 ^e^	20.59 ± 0.20 ^b^	7.72 ± 0.20 ^c^	56.17 ± 0.35 ^a^
Sea lettuce	13.35 ± 0.09 ^a^	8.85 ± 0.12 ^d^	0.63 ± 0.03 ^d^	25.70 ± 0.51 ^a^	6.49 ± 0.04 ^d^	44.99 ± 0.53 ^c^
Water silk	6.27 ± 0.07 ^e^	31.29 ± 0.02 ^a^	3.22 ± 0.15 ^a^	18.91 ± 0.32 ^c^	14.45 ± 0.09 ^a^	25.58 ± 0.50 ^e^
*Wolffia*	7.23 ± 0.06 ^d^	14.95 ± 0.02 ^c^	2.55 ± 0.31 ^b^	18.38 ± 0.14 ^d^	10.08 ± 0.43 ^b^	46.81 ± 0.47 ^b^

* Values are presented as mean ± standard deviation (n = 3). Different superscript letters in the same column indicate significant differences by the post hoc Duncan’s test at *p* < 0.05.

**Table 4:** The physical analysis of protein extract powders from different extraction methods.

Sample	Moisture (%)	a_w_	L*	a*	b*	White Index (WI)
Maceration extraction (MAE)						
Wakame	5.24 ± 0.05 ^cA^	0.18 ± 0.01 ^cA^	68.64 ± 0.28 ^cB^	−3.44 ± 0.12 ^dB^	21.54 ± 1.34 ^bcA^	61.80 ± 0.93 ^bB^
Hair seaweed	8.78 ± 0.26 ^aA^	0.38 ± 0.01 ^aA^	69.14 ± 0.54 ^cB^	2.82 ± 0.01 ^aB^	22.19 ± 0.15 ^abA^	61.88 ± 0.36 ^bB^
Sea lettuce	4.51 ± 0.05 ^dB^	0.13 ± 0.01 ^dB^	75.00 ± 0.69 ^aB^	−4.59 ± 0.04 ^eB^	20.58 ± 0.16 ^cC^	67.29 ± 0.42 ^aA^
Water silk	2.93 ± 0.01 ^eB^	0.18 ± 0.01 ^cA^	71.16 ± 0.14 ^bA^	−1.38 ± 0.03 ^cB^	16.94 ± 0.10 ^dB^	66.53 ± 0.16 ^aA^
*Wolffia*	5.52 ± 0.04 ^bB^	0.23 ± 0.01 ^bB^	54.23 ± 0.16 ^dB^	2.16 ± 0.09 ^bA^	23.30 ± 0.25 ^aA^	48.60 ± 0.24 ^cB^
Ultrasound-assisted extraction (UAE)						
Wakame	3.21 ± 0.02 ^dB^	0.11 ± 0.01 ^eB^	58.24 ± 0.37 ^bC^	1.84 ± 0.03 ^bA^	21.46 ± 0.25 ^bA^	53.01 ± 0.37 ^bC^
Hair seaweed	6.53 ± 0.14 ^bB^	0.28 ± 0.01 ^bB^	61.78 ± 0.73 ^aC^	3.57 ± 0.04 ^aA^	20.06 ± 0.20 ^cC^	56.69 ± 0.56 ^aC^
Sea lettuce	6.75 ± 0.06 ^aA^	0.34 ± 0.01 ^aA^	46.10 ± 0.77 ^cC^	−0.46 ± 0.05 ^eA^	24.74 ± 0.08 ^aA^	40.70 ± 0.67 ^cB^
Water silk	3.22 ± 0.10 ^dA^	0.17 ± 0.01 ^dB^	44.99 ± 0.30 ^dC^	0.45 ± 0.06 ^dA^	21.74 ± 0.44 ^bA^	40.84 ± 0.20 ^cC^
*Wolffia*	3.76 ± 0.04 ^cC^	0.19 ± 0.01 ^cC^	42.40 ± 0.55 ^eC^	1.18 ± 0.04 ^cB^	18.89 ± 0.37 ^dB^	39.36 ± 0.42 ^dC^
Enzyme-assisted extraction (EAE)						
Wakame	0.64 ± 0.10 ^dC^	0.10 ± 0.01 ^cB^	71.79 ± 0.53 ^bA^	−5.19 ± 0.05 ^dC^	21.66 ± 0.25 ^cA^	64.05 ± 0.28 ^cA^
Hair seaweed	1.71 ± 0.18 ^cC^	0.10 ± 0.01 ^cC^	71.29 ± 0.20 ^bA^	2.00 ± 0.01 ^bC^	20.70 ± 0.16 ^dB^	64.55 ± 0.20 ^bA^
Sea lettuce	0.79 ± 0.14 ^dC^	0.10 ± 0.01 ^cC^	76.70 ± 0.07 ^aA^	−5.40 ± 0.01 ^eC^	22.22 ± 0.04 ^bB^	67.35 ± 0.08 ^aA^
Water silk	2.47 ± 0.04 ^bC^	0.12 ± 0.01 ^bC^	56.80 ± 0.10 ^dB^	0.41 ± 0.03 ^cA^	16.88 ± 0.10 ^eB^	53.61± 0.11 ^dB^
*Wolffia*	8.82 ± 0.03 ^aA^	0.26 ± 0.01 ^aA^	59.93 ± 0.73 ^cA^	2.25 ± 0.11 ^aA^	23.06 ± 0.43 ^aA^	53.71 ± 0.42 ^dA^

Values are presented as mean ± standard deviation (n = 3). For each extraction method, the lower superscript letters within the same column indicate significant differences at *p* < 0.05, as determined by the post hoc Duncan’s test. For each raw material, capitalized letters in each column indicate significant differences across different extraction methods.

**Table 5:** Salt and protein contents of protein extract powders.

Parameters	Sample	Extraction Methods		
		MAE	UAE	EAE
Salt Content (%)	Wakame	21.56 ± 2.09 ^aB^	8.80 ± 0.24 ^bC^	24.18 ± 0.46 ^aA^
	Hair seaweed	0.02 ± 0.01 ^cC^	9.09 ± 0.10 ^aA^	4.19 ± 0.03 ^dB^
	Sea lettuce	0.02 ± 0.01 ^cC^	0.96 ± 0.03 ^dA^	0.35 ± 0.02 ^eB^
	Water silk	3.77 ± 0.02 ^bB^	1.12 ± 0.02 ^dC^	4.78 ± 0.01 ^cA^
	*Wolffia*	0.11 ± 0.01 ^cC^	2.41 ± 0.01 ^cB^	9.71 ± 0.10 ^bA^
Protein (mg/g of dried raw material)	Wakame	5.90 ± 0.32 ^cdA^	2.57 ± 0.39 ^cC^	4.33 ± 0.19 ^cdB^
	Hair seaweed	8.86 ± 0.20 ^cA^	8.98 ± 0.43 ^bA^	1.55 ± 0.20 ^dB^
	Sea lettuce	2.30 ± 0.25 ^dC^	7.32 ± 0.56 ^bA^	5.95 ± 0.45 ^cB^
	Water silk	24.25 ± 2.19 ^bA^	3.98 ± 0.55 ^cB^	24.34 ± 3.35 ^bA^
	*Wolffia*	100.79 ± 5.28 ^aA^	51.95 ± 2.66 ^aB^	51.88 ± 0.64 ^aB^

Values are presented as mean ± standard deviation (n = 3). Lowercase letter superscripts within the same column indicate significant differences as determined by the post hoc Duncan’s test at *p* < 0.05. The capitalized letter superscripts in each row indicate the significant differences among the extract materials across different extraction methods.

**Table 6:** Amino acid analysis of protein extracts from different materials and extraction methods.

Extraction Methods	Amino Acid (g/100 g)	Wakame	Hair Seaweed	Sea Lettuce	Water Silk	*Wolffia*
Maceration extraction	Asp	6.69 × 10^−2^	8.18 × 10^−2^	8.35 × 10^−2^	4.12 × 10^−2^	20.26 × 10^−2^
	Thr	1.68 × 10^−2^	3.75 × 10^−2^	2.58 × 10^−2^	1.56 × 10^−2^	4.68 × 10^−2^
	Ser	1.77 × 10^−2^	3.38 × 10^−2^	2.30 × 10^−2^	1.56 × 10^−2^	6.86 × 10^−2^
	Glu	6.83 × 10^−2^	6.87 × 10^−2^	6.37 × 10^−2^	4.37 × 10^−2^	21.70 × 10^−2^
	Gly	2.08 × 10^−2^	5.25 × 10^−2^	4.23 × 10^−2^	2.56 × 10^−2^	6.72 × 10^−2^
	Ala	2.13 × 10^−2^	4.97 × 10^−2^	4.62 × 10^−2^	2.41 × 10^−2^	49.70 × 10^−2^
	Val	1.37 × 10^−2^	2.92 × 10^−2^	2.40 × 10^−2^	1.21 × 10^−2^	10.68 × 10^−2^
	Ileu	0.80 × 10^−2^	1.88 × 10^−2^	1.09 × 10^−2^	0.57 × 10^−2^	2.55 × 10^−2^
	Lue	1.62 × 10^−2^	3.56 × 10^−2^	2.46 × 10^−2^	1.77 × 10^−2^	6.39 × 10^−2^
	Tyr	0.24 × 10^−2^	0.98 × 10^−2^	1.16 × 10^−2^	−0.00001	1.43 × 10^−2^
	Phe	0.93 × 10^−2^	2.64 × 10^−2^	1.59 × 10^−2^	0.39 × 10^−2^	3.09 × 10^−2^
	His	<24.29 × 10^−2^	<24.29 × 10^−2^	<24.29 × 10^−2^	<24.29 × 10^−2^	<24.29 × 10^−2^
	Lys	0.85 × 10^−2^	1.93 × 10^−2^	1.42 × 10^−2^	0.85 × 10^−2^	6.42 × 10^−2^
	Arg	0.54 × 10^−2^	1.81 × 10^−2^	1.90 × 10^−2^	0.67 × 10^−2^	2.72 × 10^−2^
	Pro	1.42 × 10^−2^	3.45 × 10^−2^	2.64 × 10^−2^	ND	4.92 × 10^−2^
Ultrasound-assisted extraction	Asp	1.14 × 10^−2^	18.24 × 10^−2^	11.41 × 10^−2^	3.34 × 10^−2^	26.21 × 10^−2^
	Thr	0.41 × 10^−2^	8.75 × 10^−2^	4.06 × 10^−2^	1.51 × 10^−2^	12.31 × 10^−2^
	Ser	0.48 × 10^−2^	8.49 × 10^−2^	5.51 × 10^−2^	1.47 × 10^−2^	13.20 × 10^−2^
	Glu	1.17 × 10^−2^	16.25 × 10^−2^	8.05 × 10^−2^	3.81 × 10^−2^	31.20 × 10^−2^
	Gly	0.58 × 10^−2^	11.80 × 10^−2^	5.35 × 10^−2^	1.66 × 10^−2^	14.72 × 10^−2^
	Ala	0.67 × 10^−2^	11.85 × 10^−2^	5.84 × 10^−2^	1.85 × 10^−2^	17.69 × 10^−2^
	Val	0.26 × 10^−2^	7.62 × 10^−2^	4.32 × 10^−2^	1.11 × 10^−2^	13.05 × 10^−2^
	Ileu	0.19 × 10^−2^	6.03 × 10^−2^	2.48 × 10^−2^	0.68 × 10^−2^	9.00 × 10^−2^
	Lue	0.58 × 10^−2^	10.27 × 10^−2^	6.29 × 10^−2^	2.08 × 10^−2^	24.13 × 10^−2^
	Tyr	0.17 × 10^−2^	3.14 × 10^−2^	3.33 × 10^−2^	0.58 × 10^−2^	8.12 × 10^−2^
	Phe	0.25 × 10^−2^	7.18 × 10^−2^	4.21 × 10^−2^	1.12 × 10^−2^	13.43 × 10^−2^
	His	<24.29 × 10^−2^	<24.29 × 10^−2^	<24.29 × 10^−2^	<24.29 × 10^−2^	<24.29 × 10^−2^
	Lys	0.42 × 10^−2^	5.57 × 10^−2^	3.92 × 10^−2^	1.36 × 10^−2^	14.51 × 10^−2^
	Arg	0.23 × 10^−2^	5.93 × 10^−2^	4.42 × 10^−2^	1.04 × 10^−2^	14.68 × 10^−2^
	Pro	<18.01 × 10^−2^	<18.01 × 10^−2^	<18.01 × 10^−2^	<18.01 × 10^−2^	<18.01 × 10^−2^
Enzyme-assisted extraction	Asp	9.13 × 10^−2^	3.03 × 10^−2^	16.15 × 10^−2^	9.03 × 10^−2^	19.82 × 10^−2^
	Thr	4.60 × 10^−2^	1.67 × 10^−2^	6.01 × 10^−2^	5.38 × 10^−2^	6.51 × 10^−2^
	Ser	3.12 × 10^−2^	1.13 × 10^−2^	5.91 × 10^−2^	4.51 × 10^−2^	7.82 × 10^−2^
	Glu	10.40 × 10^−2^	2.83 × 10^−2^	13.46 × 10^−2^	12.03 × 10^−2^	20.64 × 10^−2^
	Gly	5.31 × 10^−2^	2.27 × 10^−2^	7.59 × 10^−2^	5.42 × 10^−2^	7.59 × 10^−2^
	Ala	<28.96 × 10^−2^	<28.96 × 10^−2^	<28.96 × 10^−2^	<28.96 × 10^−2^	<28.96 × 10^−2^
	Val	4.81 × 10^−2^	1.45 × 10^−2^	6.30 × 10^−2^	3.41 × 10^−2^	10.13 × 10^−2^
	Ileu	2.80 × 10^−2^	1.01 × 10^−2^	3.36 × 10^−2^	2.03 × 10^−2^	4.76 × 10^−2^
	Lue	5.55 × 10^−2^	1.69 × 10^−2^	6.78 × 10^−2^	5.09 × 10^−2^	9.93 × 10^−2^
	Tyr	1.90 × 10^−2^	0.56 × 10^−2^	2.82 × 10^−2^	1.51 × 10^−2^	3.25 × 10^−2^
	Phe	2.70 × 10^−2^	1.12 × 10^−2^	4.00 × 10^−2^	2.37 × 10^−2^	4.88 × 10^−2^
	His	<24.29 × 10^−2^	<24.29 × 10^−2^	<24.29 × 10^−2^	<24.29 × 10^−2^	<24.29 × 10^−2^
	Lys	4.29 × 10^−2^	0.80 × 10^−2^	4.11 × 10^−2^	2.77 × 10^−2^	5.99 × 10^−2^
	Arg	1.50 × 10^−2^	0.40 × 10^−2^	4.44 × 10^−2^	1.29 × 10^−2^	3.89 × 10^−2^
	Pro	2.72 × 10^−2^	1.27 × 10^−2^	4.61 × 10^−2^	1.38 × 10^−2^	5.88 × 10^−2^

Note: The symbol < means “detected below MRL”, the lowest reportable concentration based on the method and calibration standards. ND means Not Detected.

**Table 7:** E-tongue analysis of the extract solutions from different materials and extraction methods.

Samples *	Sour	Bitter	Astringent	Umami	Salty	Bitter Aftertaste	Astringent Aftertaste	Umami Aftertaste
WAM	0.67	3.83	0.72	7.71	15.28	1.90	0.51	4.98
HSM	−9.36	14.22	2.97	6.48	−6.02	1.94	0.45	2.48
SLM	−4.41	5.47	3.03	8.29	7.73	0.26	0.32	5.17
WSM	0.44	6.85	3.43	12.48	31.85	4.96	1.31	3.03
WOM	−7.79	1.77	0.73	13.41	13.21	0.41	0.81	10.80
WAU	−12.93	10.51	1.98	6.69	5.45	1.60	0.06	1.68
HSU	−13.41	15.80	3.07	8.04	4.00	3.48	0.27	2.55
SLU	−19.12	13.38	2.39	4.14	−5.20	0.13	−0.12	1.12
WSU	−23.11	17.33	9.69	12.92	14.65	2.77	0.39	1.56
WOU	−17.91	8.85	3.36	11.88	7.49	0.24	0.34	1.91
WAE	0.80	3.28	0.44	8.13	16.43	2.03	0.22	6.20
HSE	−3.88	5.95	1.04	7.34	6.12	0.71	0.21	3.61
SLE	−1.27	3.93	0.23	6.33	5.33	0.39	0.05	5.19
WSE	−0.77	5.28	4.68	11.23	26.80	3.48	2.25	5.91
WOE	−4.41	1.62	1.09	11.98	16.33	1.14	1.28	8.51

* See Table 1 for abbreviations and descriptions.

## Data Availability

The raw data supporting the conclusions of this article will be made available by the authors on request.

## Supplementary Materials

The following supporting information can be downloaded at: https://www.mdpi.com/article/10.3390/foods15020305/s1. Supplementary Data S1: Analytical procedure for electronic tongue analysis.

## References

[1] Roohinejad S., Koubaa M., Barba F.J., Saljoughian S., Amid M., Greiner R. (2017). Application of Seaweeds to Develop New Food Products with Enhanced Shelf-Life, Quality and Health-Related Beneficial Properties. Food Res. Int..

[2] Tara G., Pete S., Will N., Jessica F. Food Systems and Greenhouse Gas Emissions. https://www.tabledebates.org/chapter/food-systems-and-greenhouse-gas-emissions.

[3] Nadathur S., Wanasundara J.P.D., Scanlin L. (2024). Feeding the Globe Nutritious Food in 2050: Obligations and Ethical Choices. Sustainable Protein Sources.

[4] OECD, Food and Agriculture Organization of the United Nations (2017). OECD-FAO Agricultural Outlook 2017–2026.

[5] Prosridee K., Oonsivilai R., Tira-aumphon A., Singthong J., Oonmetta-aree J., Oonsivilai A. (2023). Optimum Aquaculture and Drying Conditions for *Wolffia arrhiza* (L.) Wimn. Heliyon.

[6] Jensen S., Ólafsdóttir A., Einarsdóttir B., Hreggviðsson G.Ó., Guðmundsson H., Jónsdóttir L.B., Friðjónsson Ó.H., Jónsdóttir R. (2022). New Wave of Flavours—On New Ways of Developing and Processing Seaweed Flavours. Int. J. Gastron. Food Sci..

[7] Moerdijk-Poortvliet T.C.W., De Jong D.L.C., Fremouw R., De Reu S., De Winter J.M., Timmermans K., Mol G., Reuter N., Derksen G.C.H. (2022). Extraction and Analysis of Free Amino Acids and 5′-Nucleotides, the Key Contributors to the Umami Taste of Seaweed. Food Chem..

[8] Poojary M.M., Orlien V., Passamonti P., Olsen K. (2017). Improved Extraction Methods for Simultaneous Recovery of Umami Compounds from Six Different Mushrooms. J. Food Compos. Anal..

[9] Poojary M.M., Orlien V., Passamonti P., Olsen K. (2017). Enzyme-Assisted Extraction Enhancing the Umami Taste Amino Acids Recovery from Several Cultivated Mushrooms. Food Chem..

[10] Poojary M.M., Orlien V., Olsen K. (2019). Conventional and Enzyme-Assisted Green Extraction of Umami Free Amino Acids from Nordic Seaweeds. J. Appl. Phycol..

[11] Ummat V., Garcia-Vaquero M., Poojary M.M., Lund M.N., O’Donnell C., Zhang Z., Tiwari B.K. (2021). Green Extraction of Proteins, Umami and Other Free Amino Acids from Brown Macroalgae *Ascophyllum nodosum* and *Fucus vesiculosus*. J. Appl. Phycol..

[12] Mugavero K.L., Gunn J.P., Dunet D.O., Bowman B.A. (2014). Sodium Reduction: An Important Public Health Strategy for Heart Health. J. Public Health Manag. Pract..

[13] Yamaguchi S., Yoshikawa T., Ikeda S., Ninomiya T. (1971). Measurement of the Relative Taste Intensity of Some L-α-Amino Acids and 5′-Nucleotides. J. Food Sci..

[14] Singh R.N., Sharma S. (2012). Development of Suitable Photobioreactor for Algae Production—A Review. Renew. Sustain. Energy Rev..

[15] Cole A.J., De Nys R., Paul N.A. (2015). Biorecovery of Nutrient Waste as Protein in Freshwater Macroalgae. Algal Res..

[16] Černá M. (2011). Seaweed Proteins and Amino Acids as Nutraceuticals. Advances in Food and Nutrition Research.

[17] Cherry P., O’Hara C., Magee P.J., McSorley E.M., Allsopp P.J. (2019). Risks and Benefits of Consuming Edible Seaweeds. Nutr. Rev..

[18] Sitthiwong N. (2019). Pigment and Nutritional Value of *Spirogyra* spp. in Sakon Nakhon, Nakhon Phanom and Mukdahan Provinces. Prog. Appl. Sci. Technol..

[19] Sánchez-Machado D.I., López-Cervantes J., López-Hernández J., Paseiro-Losada P. (2004). Fatty Acids, Total Lipid, Protein and Ash Contents of Processed Edible Seaweeds. Food Chem..

[20] Appenroth K.-J., Sree K.S., Bog M., Ecker J., Seeliger C., Böhm V., Lorkowski S., Sommer K., Vetter W., Tolzin-Banasch K. (2018). Nutritional Value of the Duckweed Species of the Genus *Wolffia* (Lemnaceae) as Human Food. Front. Chem..

[21] Bhanthumnavin K., Mcgarry M.G. (1971). *Wolffia arrhiza* as a Possible Source of Inexpensive Protein. Nature.

[22] Figueroa V., Bunger A., Ortiz J., Aguilera J.M. (2022). Sensory Descriptors for Three Edible Chilean Seaweeds and Their Relations to Umami Components and Instrumental Texture. J. Appl. Phycol..

[23] Haroun A.A., Matazu I.K., Abdulhamid Y., Sani J. (2019). Molecular Identification of Green Algae, Spirogyra Porticalis, along Parts of River Kaduna and Its Potential for Singlecell Protein (SCP) Production. Niger. Ann. Pure Appl. Sci..

[24] Koli D., Rudra S., Bhowmik A., Pabbi S. (2022). Nutritional, Functional, Textural and Sensory Evaluation of Spirulina Enriched Green Pasta: A Potential Dietary and Health Supplement. Foods.

[25] Peñalver R., Lorenzo J.M., Ros G., Amarowicz R., Pateiro M., Nieto G. (2020). Seaweeds as a Functional Ingredient for a Healthy Diet. Mar. Drugs.

[26] Zhao Y., Zhang M., Devahastin S., Liu Y. (2019). Progresses on Processing Methods of Umami Substances: A Review. Trends Food Sci. Technol..

[27] Chemat F., Rombaut N., Sicaire A.-G., Meullemiestre A., Fabiano-Tixier A.-S., Abert-Vian M. (2017). Ultrasound Assisted Extraction of Food and Natural Products. Mechanisms, Techniques, Combinations, Protocols and Applications. A Review. Ultrason. Sonochem..

[28] Khoddami A., Wilkes M., Roberts T. (2013). Techniques for Analysis of Plant Phenolic Compounds. Molecules.

[29] Wijesinghe W.A.J.P., Jeon Y.-J. (2012). Enzyme-Assistant Extraction (EAE) of Bioactive Components: A Useful Approach for Recovery of Industrially Important Metabolites from Seaweeds: A Review. Fitoterapia.

[30] United Nations Department of Economic and Social Affairs The Sustainable Development Goals Report 2025. https://unstats.un.org/sdgs/report/2025/.

[31] AOAC (2000). Official Methods of Analysis of AOAC International.

[32] Purdi T.S., Setiowati A.D., Ningrum A. (2023). Ultrasound-Assisted Extraction of Spirulina Platensis Protein: Physicochemical Characteristic and Techno-Functional Properties. J. Food Meas. Charact..

[33] Rodrigues D., Sousa S., Silva A., Amorim M., Pereira L., Rocha-Santos T.A.P., Gomes A.M.P., Duarte A.C., Freitas A.C. (2015). Impact of Enzyme- and Ultrasound-Assisted Extraction Methods on Biological Properties of Red, Brown, and Green Seaweeds from the Central West Coast of Portugal. J. Agric. Food Chem..

[34] Vilg J.V., Undeland I. (2017). pH-Driven Solubilization and Isoelectric Precipitation of Proteins from the Brown Seaweed *Saccharina latissima*—Effects of Osmotic Shock, Water Volume and Temperature. J. Appl. Phycol..

[35] Lima M.E.P., Carneiro M.E., Nascimento A.E., Grangeiro T.B., Holanda M.L., Amorim R.C.N., Benevides N.M.B. (2005). Purification of a Lectin from the Marine Red Alga *Gracilaria cornea* and Its Effects on the Cattle Tick *Boophilus microplus* (Acari: Ixodidae). J. Agric. Food Chem..

[36] Juel N., Juul L., Tanambell H., Dalsgaard T.K. (2024). Extraction and Purification of Seaweed Protein from Ulva Sp.—Challenges to Overcome. LWT.

[37] Duangjarus N., Chaiworapuek W., Rachtanapun C., Ritthiruangdej P., Charoensiddhi S. (2022). Antimicrobial and Functional Properties of Duckweed (*Wolffia globosa*) Protein and Peptide Extracts Prepared by Ultrasound-Assisted Extraction. Foods.

[38] Susi S., Al Hakim H.M. (2022). Comparison of NaCl Levels in Seasoning Powder Formulation of Nagara Bean Flour (*Vigna unguiculata* Ssp. *cylindrica*) and Oyster Mushroom Using the Mohr Method by Direct Titration and Ash Mineral Titration. J. Appl. Food Technol..

[39] Lowry O.H., Rosebrough N.J., Farr A.L., Randall R.J. (1951). Protein Measurement with The Folin Phenol Reagent. J. Biol. Chem..

[40] Chen Y., Hong J., Jiang Z., Wu L., Wang X., Zhu Y., Jiang Z., Ni H., Zheng M. (2023). Effect of Red Algae Powder on Gel Properties and In Vitro Hypolipidemic Activity of Fish Balls. Algal Res..

[41] Qin Y., Pillidge C., Harrison B., Adhikari B. (2025). Development and Characterization of Soy Protein-Based Custard-like Soft Foods for Elderly Individuals with Swallowing Difficulties. Food Res. Int..

[42] (2005). Animal Feeding Stuffs—Determination of Amino Acids Content.

[43] Peter N.R., Raja N.R., Rengarajan J., Radhakrishnan Pillai A., Kondusamy A., Saravanan A.K., Changaramkumarath Paran B., Kumar Lal K. (2024). A Comprehensive Study on Ecological Insights of *Ulva lactuca* Seaweed Bloom in a Lagoon along the Southeast Coast of India. Ocean Coast. Manag..

[44] Taboada M.C., Millán R., Miguez M.I. (2013). Nutritional Value of the Marine Algae Wakame (*Undaria pinnatifida*) and Nori (*Porphyra purpurea*) as Food Supplements. J. Appl. Phycol..

[45] Yongkhamcha B., Buddhakala N. (2023). Phytochemical Compositions, Nutritional Contents, Cytotoxicity and Anti-Inflammatory Activity of Different Extracts from *Spirogyra neglecta* (Hassall) Kützing. Trends Sci..

[46] Mercer D.G. (2008). Solar Drying in Developing Countries: Possibilities and Pitfalls. Using Food Science and Technology to Improve Nutrition and Promote National Development.

[47] Rao Q., Fisher M.C., Guo M., Labuza T.P. (2013). Storage Stability of a Commercial Hen Egg Yolk Powder in Dry and Intermediate-Moisture Food Matrices. J. Agric. Food Chem..

[48] Sablani S.S., Kasapis S., Rahman M.S. (2007). Evaluating Water Activity and Glass Transition Concepts for Food Stability. J. Food Eng..

[49] Pathare P.B., Opara U.L., Al-Said F.A.-J. (2013). Colour Measurement and Analysis in Fresh and Processed Foods: A Review. Food Bioprocess Technol..

[50] Soimaloon P., Tinchan P., Horng-Liang L. (2018). Effect of Extraction Conditions on Color, pH, Flavor Profile and Ribonucleotide Contents of *Limnophila aromatica* (Lam.) Merr. Extracts. Appl. Sci. Eng. Prog..

[51] Yong S.X.M., Song C.P., Choo W.S. (2021). Impact of High-Pressure Homogenization on the Extractability and Stability of Phytochemicals. Front. Sustain. Food Syst..

[52] Patel A.K., Albarico F.P.J.B., Perumal P.K., Vadrale A.P., Nian C.T., Chau H.T.B., Anwar C., Wani H.M.U.D., Pal A., Saini R. (2022). Algae as an Emerging Source of Bioactive Pigments. Bioresour. Technol..

[53] Torres P., Santos J.P., Chow F., Dos Santos D.Y.A.C. (2019). A Comprehensive Review of Traditional Uses, Bioactivity Potential, and Chemical Diversity of the Genus *Gracilaria* (Gracilariales, Rhodophyta). Algal Res..

[54] Pagliuso D., Grandis A., Fortirer J.S., Camargo P., Floh E.I.S., Buckeridge M.S. (2022). Duckweeds as Promising Food Feedstocks Globally. Agronomy.

[55] Gómez Barrio L.P., Cabral E.M., Zhao M., Álvarez García C., Senthamaraikannan R., Padamati R.B., Tiwari U., Curtin J.F., Tiwari B.K. (2022). Comparison Study of an Optimized Ultrasound-Based Method versus an Optimized Conventional Method for Agar Extraction, and Protein Co-Extraction, from *Gelidium sesquipedale*. Foods.

[56] Lian H., Wen C., Zhang J., Feng Y., Duan Y., Zhou J., He Y., Zhang H., Ma H. (2021). Effects of Simultaneous Dual-Frequency Divergent Ultrasound-Assisted Extraction on the Structure, Thermal and Antioxidant Properties of Protein from *Chlorella pyrenoidosa*. Algal Res..

[57] Adam F., Abert-Vian M., Peltier G., Chemat F. (2012). “Solvent-Free” Ultrasound-Assisted Extraction of Lipids from Fresh Microalgae Cells: A Green, Clean and Scalable Process. Bioresour. Technol..

[58] Bojorges H., López-Rubio A., Martínez-Abad A., Fabra M.J. (2023). Overview of Alginate Extraction Processes: Impact on Alginate Molecular Structure and Techno-Functional Properties. Trends Food Sci. Technol..

[59] Lin J., Jiao G., Kermanshahi-pour A. (2022). Algal Polysaccharides-Based Hydrogels: Extraction, Synthesis, Characterization, and Applications. Mar. Drugs.

[60] Raja K., Kadirvel V., Subramaniyan T. (2022). Seaweeds, an Aquatic Plant-Based Protein for Sustainable Nutrition—A Review. Future Foods.

[61] Synytsya A., Čopíková J., Kim W.J., Park Y.I., Kim S.-K. (2015). Cell Wall Polysaccharides of Marine Algae. Springer Handbook of Marine Biotechnology.

[62] De Souza Celente G., Sui Y., Acharya P. (2023). Seaweed as an Alternative Protein Source: Prospective Protein Extraction Technologies. Innov. Food Sci. Emerg. Technol..

[63] Naseri A., Marinho G.S., Holdt S.L., Bartela J.M., Jacobsen C. (2020). Enzyme-Assisted Extraction and Characterization of Protein from Red Seaweed *Palmaria palmata*. Algal Res..

[64] Malvis Romero A., Picado Morales J.J., Klose L., Liese A. (2023). Enzyme-Assisted Extraction of Ulvan from the Green Macroalgae *Ulva fenestrata*. Molecules.

[65] Perez-Vazquez A., Carpena M., Barciela P., Cassani L., Simal-Gandara J., Prieto M.A. (2023). Pressurized Liquid Extraction for the Recovery of Bioactive Compounds from Seaweeds for Food Industry Application: A Review. Antioxidants.

[66] Ruekaewma N., Piyatiratitivorakul S., Powtongsook S. (2015). Culture System for *Wolffia globosa* L. (Lemnaceae) for Hygiene Human Food. Songklanakarin J. Sci. Technol..

[67] Zhao X., Wei Y., Gong X., Xu H., Xin G. (2020). Evaluation of Umami Taste Components of Mushroom (*Suillus granulatus*) of Different Grades Prepared by Different Drying Methods. Food Sci. Hum. Wellness.

[68] Seo W.H., Lee H.G., Baek H.H. (2008). Evaluation of Bitterness in Enzymatic Hydrolysates of Soy Protein Isolate by Taste Dilution Analysis. J. Food Sci..

[69] Sun X., Zheng J., Liu B., Huang Z., Chen F. (2022). Characteristics of the Enzyme-Induced Release of Bitter Peptides from Wheat Gluten Hydrolysates. Front. Nutr..

[70] Mouritsen O.G., Duelund L., Petersen M.A., Hartmann A.L., Frøst M.B. (2019). Umami Taste, Free Amino Acid Composition, and Volatile Compounds of Brown Seaweeds. J. Appl. Phycol..

