# Supplementary material for: Impact of Green Extraction Methods for Algae and Aquatic Plants on Amino Acid Composition and Taste Detection Using Electronic Tongue Analysis
Source: Foods. 2026 Jan 14;15(2):305. doi: 10.3390/foods15020305 (PMC12840424; doi:10.3390/foods15020305)
Supplement: Supplementary file 1 [file foods-15-00305-s001.zip › foods-4042714-supplementary.pdf]

Article

Impact of Green Extraction Methods for Algae and Aquatic Plants on Amino Acid Composition and Taste Detection Using Electronic Tongue Analysis

Lyket Chuon [1], Witoon Prinyawiwatkul [2], Amporn Sae-Eaw [1] and Peerapong Wongthahan [1,*]

1 Department of Food Technology, Faculty of Technology, Khon Kaen University, Khon Kaen 40002, Thailand; lyket.c@kkumail.com (L.C.); sampor@kku.ac.th (A.S.-E.)

2 School of Nutrition and Food Sciences, Agricultural Center, Louisiana State University, Baton Rouge LA 70803, USA; wprinya@lsu.edu

* Correspondence: peerwo@kku.ac.th

Supplementary data

Analytical procedure (Electronic tongue analysis)

1. Sensor and electrode preconditioning

Prior to analysis, six taste sensors (AAE, CT0, CA0, UM2, C00, and AE1) and the Ag/AgCl reference electrode for inner and outer arms were preconditioned for at least 24 h using the manufacturer-provided reference solution, while ensuring that the internal solution of each sensor was filled to the recommended level. This procedure was performed to stabilize the lipid/polymer membranes and ensure consistent baseline responses before use.

2. Measurement maintenance (MM)

- The taste sensors and Ag/AgCl reference electrodes were connected to the corresponding measurement sensor head and operated using sensor arm. Measurement maintenance, including sensor rinsing, baseline stabilization, and system performance checks, was conducted according to the manufacturer's standard operating procedures to ensure stable instrument operation throughout the analysis.
- In this step, sensor calibration and quality control were verified by preconditioning the sensors and monitoring baseline stability using manufacturer-provided reference solutions and standard samples including salty, sour, umami, bitter (+), bitter (−), and astringent samples, prior to sample measurement.
- The measurement was performed for 5 cycles.

3. Sample measurement

- Extraction samples were prepared and visually inspected to ensure the absence of particles or precipitates, then equilibrated to room temperature prior to analysis.
- pH and conductivity of each sample were measured to confirm suitability for electronic tongue measurement, within the ranges of pH 2-8 and 1-10 mS/cm, respectively.
- Samples and reference (washing) solutions were transferred into measurement cups at the appropriate fill level and arranged according to the predefined measurement sequence. Each sample was measured four times, with the first measurement excluded as a stabilization run, and the remaining three measurements averaged for data analysis.

- Three extraction methods were evaluated- maceration extraction, ultrasound-assisted extraction, and enzyme-assisted extraction-across five different species. The measurement order was arranged as Wakame, Hair seaweed, Sea lettuce, Water silk, and *Wolffia*.

4. Sensor performance checking
    - Sensor performance checking was conducted to ensure proper sensor function after replacement of the washing solution and prior to sample analysis.
    - Sensor stability and functionality were verified by repeated measurements of the reference solution, and the system automatically evaluated sensor responses as “pass” or “not pass” according to the manufacturer’s criteria. Only sensors that met the “pass” criteria were used for sample analysis. If a “not pass” result was obtained, sensor quality was checked by confirming the absence of air bubbles and ensuring that the internal solution level was appropriate before re-checking.

5. Data processing and chemometric analysis
    - Sensor output data were exported from the management server and processed using XLSTAT 2025.1.3. Chemometric analysis was conducted using principal component analysis (PCA) and hierarchical cluster analysis (HCA) to evaluate differences in taste profiles and to investigate relationships between electronic tongue responses and amino acid composition.
